# The role of circular RNA in Diabetic Nephropathy

**DOI:** 10.7150/ijms.71648

**Published:** 2022-05-20

**Authors:** Chao Tu, Liangzhi Wang, Lan Wei, Zhuyan Jiang

**Affiliations:** 1 Department of Internal Medicine, The Third Affiliated Hospital of Soochow University, Changzhou, Jiangsu, 213000, China; 2 Department of Dermatology, Southwest Hospital, Army Military Medical University, Chongqing, 400038, China

**Keywords:** diabetic nephropathy, circular RNAs, biomarker, mesangial cells, tubule cells

## Abstract

Diabetic nephropathy (DKD) is the most common chronic microvascular complication of diabetes. About 20%-40% of diabetics develop DKD, which eventually leads to chronic kidney failure. Although progress has been made in diagnosis and treatment tools, diabetic nephropathy is still a major clinical problem. In recent years, circular RNA (CircRNA) has become a research hotspot. CircRNA is a non-coding RNA formed by covalently closing the 5 'and 3' ends of the precursor RNA. CircRNA has powerful biological functions. CircRNA can regulate the expression of target genes through competitive binding with microRNA, thus playing the biological role of endogenous RNA (CeRNA). Many studies have shown that circRNAs plays an important role in malignant tumors, autoimmune system diseases, coronary heart disease and other diseases. More and more studies have shown that it can also be used as a biomarker of diabetes and diabetic nephropathy. This review summarizes the origin, classification, biogenesis and regulatory mechanisms of circRNAs. In addition, the pathogenesis and clinical significance of circRNAs as competing endogenous RNAs involved in diabetic nephropathy were also introduced. This will help us fully understand the pathological mechanism of diabetic nephropathy and develop new therapeutic targets or treatment options to improve the prognosis of patients with diabetic nephropathy.

## Introduction

Diabetes is one of the common metabolic disorders that seriously affect people's quality of life and health. Diabetes is characterized by insulin resistance and impaired glucose tolerance. The causes of diabetes are related to many factors, such as environmental factors, genetic factors, lifestyle factors, inflammatory factors, disorders of glucose and lipid metabolism, dietary habits 1. Complications of diabetes include microvascular disease such as chronic renal failure, retinopathy changes and peripheral neuropathy, macrovascular diseases such as coronary atherosclerosis, peripheral arterial disease, cerebral infarction and other extremely harmful complications. Diabetic nephropathy is a common chronic microvascular complication of diabetes, which eventually leads to chronic renal failure 2. About 20%-40% of people with diabetes develop diabetic nephropathy, depending on population and race. Early clinical diagnosis of diabetic nephropathy is usually based on microalbuminuria (30-300 mg/day) or urinary albumin/creatinine ratio (>30mg/g creatinine) 3. Although progress has been made in diagnosis and treatment tools, diabetic nephropathy still brings huge physical and economic burden to patients, seriously endangering their quality of life, so it is very important to find new treatment targets. CircRNAs are a new type of non-coding RNA, which is considered to be a by-product of transcription and has no function 4. However, in recent years, CircRNAs have been identified as an important regulator of many diseases, such as cancer, cardiovascular diseases and autoimmune diseases 5. In addition, CircRNAs have attracted more and more attention in recent years because of its role in the development of many renal diseases such as renal cell carcinoma, acute renal damage, chronic glomerulonephritis and diabetic renal injury. However, the expression profile of CircRNAs in diabetic nephropathy is not clear. Some studies have shown that CircRNAs can be detected in blood and kidney cells of patients with diabetic nephropathy 6. This article reviews the origin, classification and specific biological functions of circRNAs. At the same time, their specific regulatory mechanism, clinical value and therapeutic significance in diabetic nephropathy were analyzed.

## Pathogenesis of Diabetic Nephropathy

**Figure 1:**
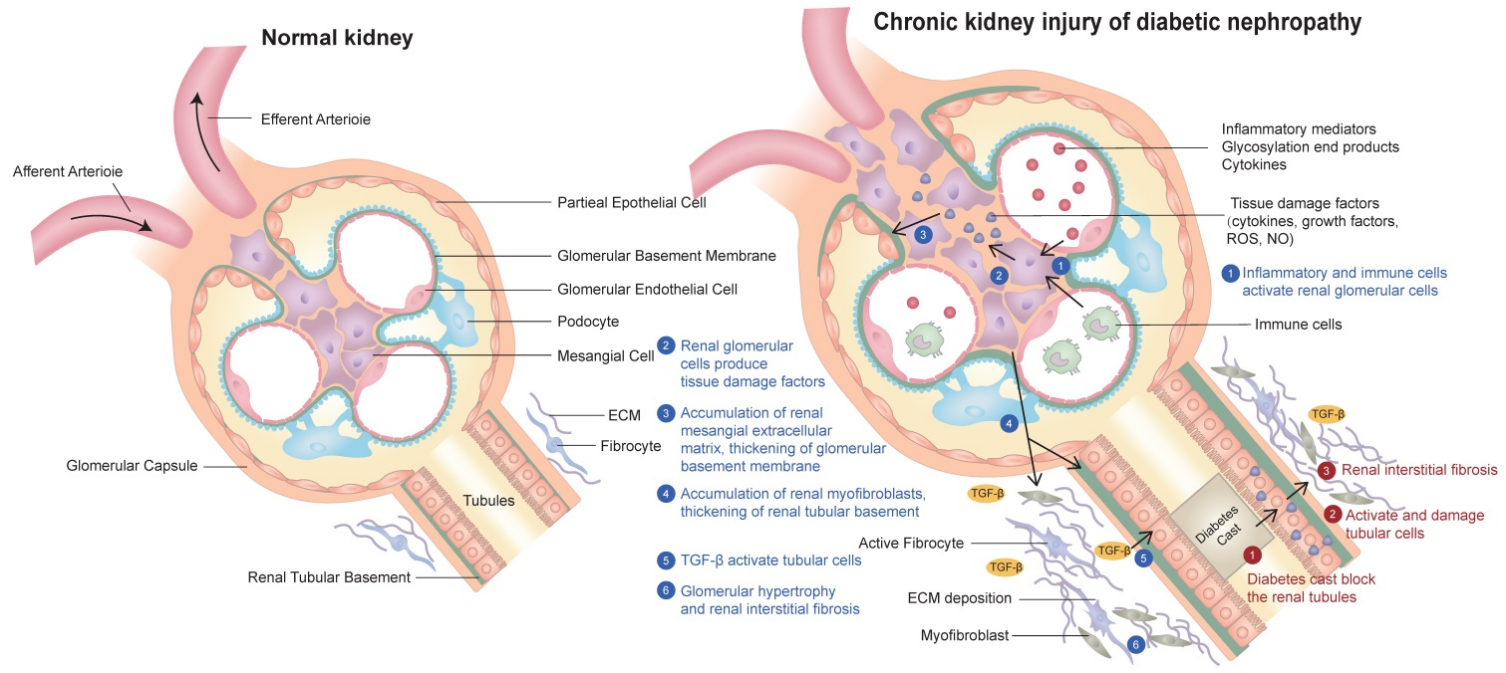
Schematic diagram of the pathogenesis of chronic kidney injury in diabetic nephropathy in this review:(1) Inflammation and immune cells activate glomerular cells; (2) Glomerular cells produce various tissue damage factors; (3) The extracellular matrix (ECM) of glomerular mesangial cells increases and proliferates; (4) Tubular basement membrane thickening and tubular myofibroblast accumulation; (5) TGF-β activates renal tubular cells; (6) Glomerular hypertrophy and renal interstitial fibrosis.

Diabetic nephropathy is the most common complication of diabetes. The clinical manifestations of DKD are varied. The common symptoms are foamy urine, eyelid edema, frequent urination, nausea, vomiting and loss of appetite. The pathogenesis of diabetic nephropathy has the following characteristics: (1) inflammatory mediators, advanced glycation end products and cytokines act on Mesangial cells and secrete various bioactive substances, resulting in the proliferation of extracellular matrix (ECM) of Mesangial cells. In addition, renal tubular epithelial cells, podocytes, macrophages and mesenchymal stem cells derived from adipose tissue can also secrete exosomes, resulting in glomerular fibrosis and sclerosis^7^.They all participate in the pathophysiological process and therapeutic target of DKD. (2) Inflammatory mediators act on glomerular intrinsic cells to produce reactive oxygen species and inflammatory factors, which further damage glomerular intrinsic cells and podocytes and cause renal inflammation. As a result, inflammation further causes glomerular Mesangial cells to produce too much Mesangial matrix, affecting renal tubular epithelial cells, resulting in tubulointerstitial fibrosis and sclerosis 8. (3) Diabetes leads to the deposition of urinary protein in the mesangial area, which damages the mesangial cells.This also leads to damage to mesangial cells, increased mesangial cell proliferation and mesangial matrix production, resulting in hyalinization of glomerular afferent arterioles, further leading to glomerular fibrosis and sclerosis 9. (4) Diabetic patients usually produce proteinuria through specific molecular pathways, including renin-angiotensin-aldosterone system, protein kinase C, transforming growth factor β1, monocyte chemical protein-1, up-regulated plasminogen activator inhibitor-1, connective tissue growth factor, collagen and cytokines. Because urinary protein increases the reabsorption of protein in renal tubules and promotes the production of ammonia, the formation of ammonia will aggravate renal tubulointerstitial fibrosis and sclerosis, leading to the formation of diabetic nephropathy 10. (5) Hyperglycemia can promote the secretion of fibronectin, type I collagen, type IV collagen, interleukin-6, interleukin-1β and tumor necrosis factor-α, resulting in oxidative stress, apoptosis, injury of renal tubular epithelial cells and glomerular and tubular fibrosis, and accelerate the occurrence and development of DKD. The typical pathological features of diabetic nephropathy are proteinuria, accumulation of extracellular matrix, glomerular hypertrophy, Mesangial matrix and glomerular basement membrane thickening, glomerular and tubular fibrosis and sclerosis 6, 11. (A schematic diagram of the pathogenesis of diabetic nephropathy is shown in Figure 1).

## Classification, biogenesis and regulatory mechanism of CircRNAs

Many mammalian genomes can be transcribed into noncoding RNA, usually by RNA polymerase II. Noncoding RNAs are generally divided into two types based on their size: small noncoding RNAs, including microribonucleic acids (miRNAs) and some circRNAs. Large non-coding RNAs, including long non-coding RNAs, are usually divided into linear RNAs and circRNAs 12. CircRNAs is a kind of single-stranded RNA molecule with covalent closed structure. These RNA are relatively stable in body fluids and resistant to exonuclease, with an average half-life of 48 hours in plasma. CircRNAs are conserved and tissue specific, and they can be highly concentrated in cells 13. CircRNAs can be produced by introns or intergenomic regions of genes. They are highly stable because they have no 5 'cap and 3' tail, nor do they contain polyadenosine tails. CircRNA is produced by reverse splicing because most CircRNA is produced after the transcription of their parents' genes is completed 14. CircRNA is mainly found in the nucleus. They are generally divided into three types: exon CircRNA (EcRNA), which contains one or more exons from alternative splicing; intron CircRNA (CiRNA), which contains only introns; and exon-intron CircRNA, which contains both exons and introns 15.

**Figure 2:**
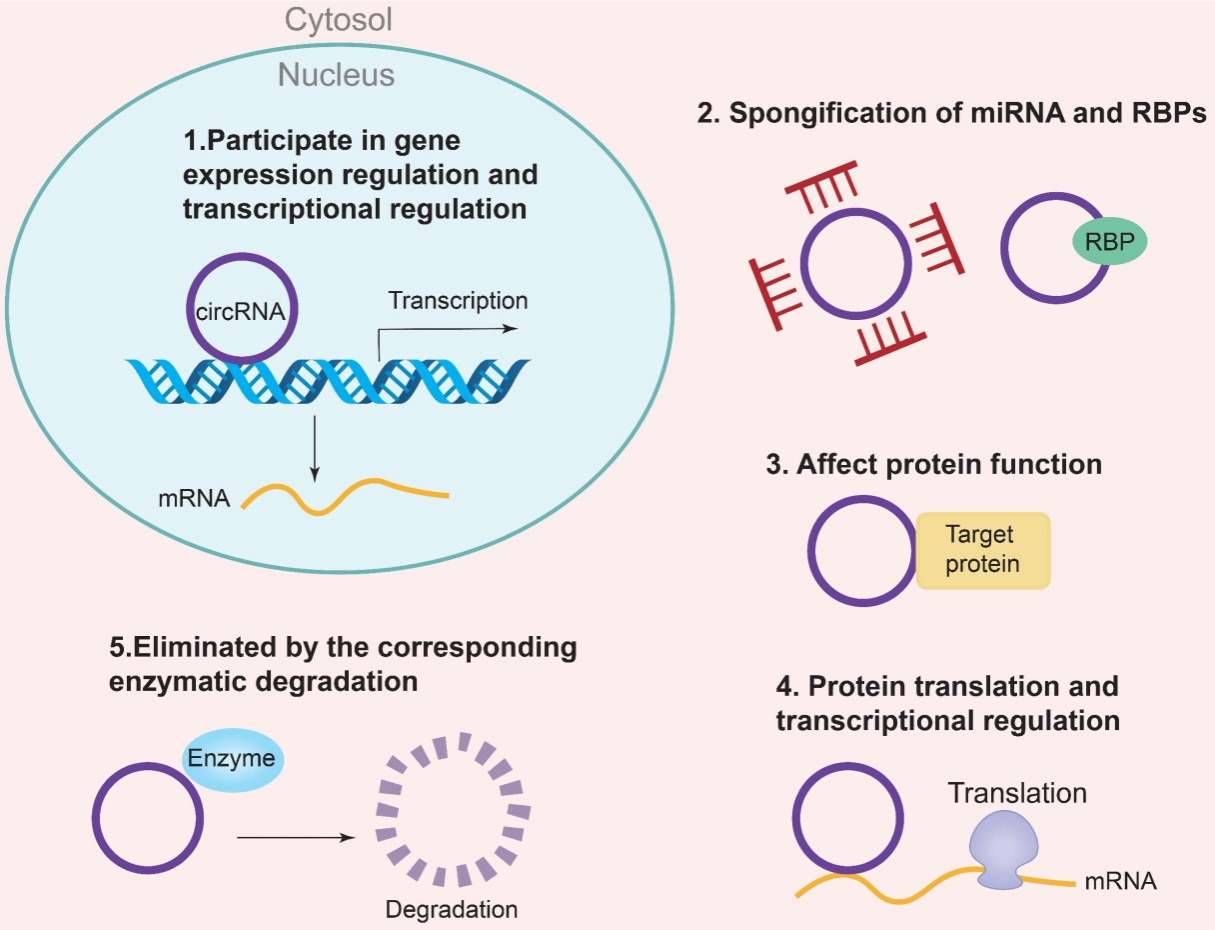
Biological functions of CircRNA. (1) Participating in gene expression regulation and transcriptional regulation; (2) Spongification of miRNA and RBPs; (3) Affecting protein function; (4) protein translation and transcriptional regulation; (5) Eliminated by the correspongding enzymatic degradation.

The biogenesis of CircRNAs generally includes exon lasso, ALU reverse splicing and reverse repetitive complementation, RNA binding protein regulation and so on. Currently, there are five specific mechanisms to regulate the molecular function of CircRNA 16: (1) Regulation of gene expression and transcription; (2) Sponging of miRNAs and RNA-binding proteins (RBPs); (3) Regulation of protein function; (4) Regulation of protein translation and transcription; (5) Degradation by specific enzymes Eliminate circRNAs. In addition, circRNAs are also involved in the regulation of gene expression, microRNA (miRNA) repression, RNA-binding proteins, and nuclear transcription 17. The regulatory mechanism of CircRNAs is the CeRNA mechanism, which can act as a competitive endogenous ribonucleic acid sponge to inhibit miRNA, resulting in the inability of mRNA to bind to the target DNA. The result is an increase in RNA and fine-tuning the expression of related genes at the post-transcriptional level. In addition, the corresponding pseudogenes produced by CircRNA can be integrated into some genomes or degraded by ribonuclease and removed from cells 18, 19. The specific mechanism of CircRNAs biogenesis is shown in Figure 2. Some studies have shown that CircRNA may be a biomarker of diabetic microangiopathy and a new therapeutic target. Therefore, the biogenesis, classification and function of CircRNA can become a new research field of human diseases including diabetes and its DKD.

## CircRNAs are involved in the pathogenesis of diabetic nephropathy

CircRNA is formed by reverse splicing of exons. The structure of CircRNA is relatively stable because they are long non-coding RNA without 5 'and 3' ends, and only a small amount of CircRNA can be translated into small peptides 20. CircRNA is often used as a sponge of microRNA to reduce biological activity, mainly through competitive interaction with target genes, resulting in up-regulation or down-regulation of target gene expression. CircRNA-miRNA gene axis plays an important role in the pathogenesis of many diseases. Studies have shown that CircRNA may be a promising biomarker and valuable clinical treatment target for the diagnosis of diabetes and diabetic nephropathy. An et al. found that there is a significant relationship between CircRNA and microangiopathy caused by diabetes 21. However, there are few studies on the expression profile of CircRNA in diabetic nephropathy. Therefore, CircRNA has become a new research field of diabetic nephropathy.

**Figure 3:**
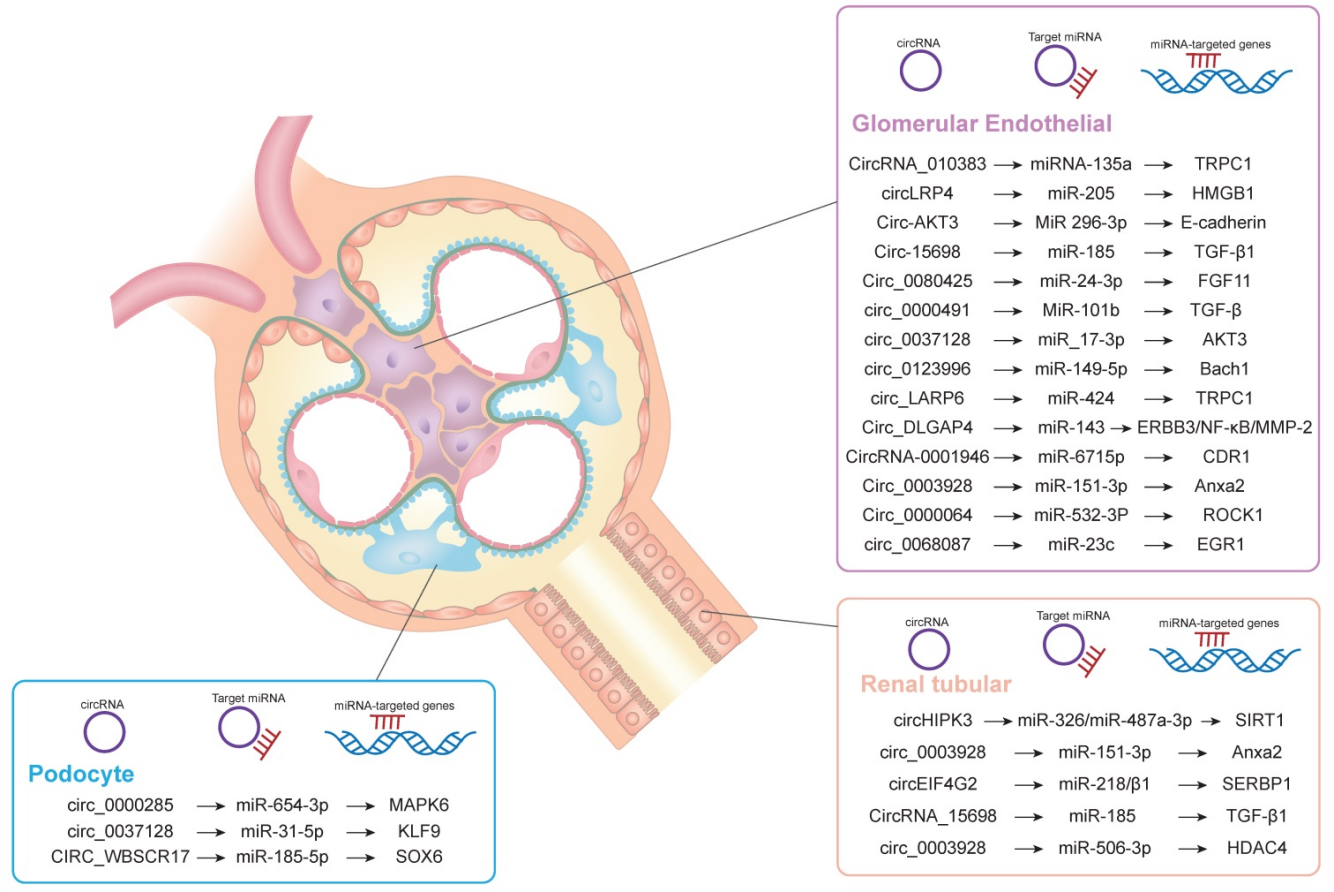
Schematic diagram of circrnas participating in DKD through ceRNA mechanism. CircRNA is involved in the pathogenesis of DKD through ceRNA mechanism in podal process cells, glomerular endothelial cells and renal tubules.

Diabetic nephropathy is the most common cause of chronic renal failure. It is still the main cause of morbidity and mortality in patients with diabetes, which can cause great harm and poor prognosis. At present, the clinical criteria for the diagnosis of DKD are decreased glomerular filtration rate, microalbuminuria (30-300mg/d), increased urinary albumin/creatinine ratio (>30mg/g) and increased 24-hour urinary protein level 22. The pathological features of DKD are proteinuria, glomerular ECM accumulation, glomerular hypertrophy, Mesangial cell proliferation and hypertrophy, Mesangial matrix and glomerular basement membrane thickening and renal fibrosis 23. The specific pathological mechanism of DKD includes that inflammatory mediators act on glomerular intrinsic cells to produce reactive oxygen species, which further damage glomerular Mesangial cells and cause renal inflammation. In addition, glomerular Mesangial cells produce additional Mesangial matrix, which acts on renal tubular epithelial cells, resulting in tubulointerstitial fibrosis and sclerosis. In addition, tissue damage factors such as cytokines, growth factors and inflammatory factors can also cause the accumulation of renal myofibroblasts, lead to glomerular and tubular basement membrane thickening, and further promote glomerular hypertrophy and renal interstitial fibrosis. This process is generally irreversible 24. Studies have shown that CircRNA plays an important role in diabetic inflammation and renal tubular fibrosis, and the degree of inflammation is positively correlated with the development stage of diabetic nephropathy 25. Wu et al. found that circRNAs are involved in the progression of diabetic nephropathy. Simultaneously promotes mesangial cell proliferation and fibrosis 26. Therefore, CircRNA may become a new clinical target for the diagnosis and treatment of diabetic nephropathy. However, there are few reports on the relationship between CircRNA and diabetic nephropathy. Therefore, this review focuses on the correlation between circRNA and DKD. The kidneys are generally divided into glomeruli and tubules. Glomeruli can be further divided into mesangial cells and podocytes 27. Currently, circRNA evidence for diabetic nephropathy mainly comes from tubular epithelial cells and mesangial cells. CircRNAs inhibit the activity of corresponding RNA-binding proteins mainly by inhibiting the release of sponge miRNAs. Thereby induces mesangial cell proliferation and interstitial fibrosis. In addition, hyperglycemia, inflammatory cell apoptosis and oxidative stress also promote the formation of diabetic nephropathy 28, 29. Figure 3 shows the role of CircRNAs in the pathogenesis of diabetic nephropathy.

## The role of CircRNAs in Diabetic Nephropathy

CircRNAs is a new regulatory factor in the occurrence and development of diabetic nephropathy. Li et al. suggest that CircRNA-0001946 may be one of the targets for the treatment of diabetic nephropathy by inhibiting the activation of miR-0001946/miR-6715p/CDR1 gene axis 30. Other studies have found that CircRNA_15698, as a sponge of miR-185, inhibits its expression, and then increases the expression of transforming growth factor-β1 protein, leading to the accumulation of glomerular extracellular matrix.This suggests that the CircRNA_15698/miR-185/ transforming growth factor-β1 gene axis can lead to the accumulation of extracellular matrix in Mesangial cells and renal fibrosis, resulting in diabetic nephropathy 31. Liu et al. showed that the expression of CIRC_0080425 and miR-24-3p induced the expression of fibroblast growth factor-11, and its expression level correlated with the severity of diabetic nephropathy. In addition, they found that CIRC_0080425 promoted the proliferation and fibrosis of mesangial cells, leading to the subsequent progression of diabetic nephropathy 32. The deletion of CircRNA_010383 promotes the expression of TRPC1 protein through the sponge of miRNA-135a, which leads to the formation of DKD proteinuria, the proliferation of glomerular Mesangial cells and renal fibrosis and sclerosis 33. CircRNA_010383 can inhibit the synthesis of proteins in glomerular mesangial cells and extracellular matrix of renal tubular epithelium, thereby slowing the progression of diabetic nephropathy through circRNA_010383. CircLRP6, acting as a sponge of miR-205, can regulate the accumulation of ECM in mesangial cells and reduce the oxidative inflammatory response, thereby reducing the progression of DKD disease 34. CircACTR2 plays a role in the inflammatory response in DKD tissues, as well as the proliferation and necrosis of glomerular and tubular epithelial cells. It also plays a key role in the regulation of renal fibrosis. The deletion of CircACTR2 reduces the production of pyrophosphate cells, interleukin-1β, type IV collagen and fibronectin, thus reducing the occurrence and development of renal tubular fibrosis 35.

Inflammation due to oxidative stress and the release of inflammatory factors is an important mechanism leading to diabetic nephropathy. Inflammation-induced podocyte injury is a major cause of glomerular fibrosis, sclerosis, and progressive loss of renal function 36. Therefore, studying the mechanism of podocyte injury is a new target for the treatment of DKD. Through the CIRC_0000285/miR-654-3p/MAPK6 mechanism, inflammatory cytokines are released and increase the damage of DKD podocytes, which leads to the further progression of DKD disease. In addition, CIRC_0000491/miR-101b/transforming growth factor β-RI gene axis can also induce the synthesis of glomerular extracellular matrix and renal fibrosis-related proteins in patients with diabetic nephropathy, which will also lead to further aggravation of DKD 37. Hsa_Circ_0003928 binds to miR-151-3p and induces inflammation and cell injury by regulating the expression of Anxa2 protein. Therefore, down-regulation of hsa_Circ_0003928 can reduce glomerular cell injury and inflammation caused by hyperglycemia, thus reducing the progression of DKD. Circ_0037128 is highly expressed in podocytes induced by hyperglycemia and diabetic nephropathy. Circ_0037128 acts as a sponge for miR_17-3p, inhibiting the expression of miR_17-3p, leading to increased AKT_3 synthesis, proliferation and fibrosis of mesangial cells, and ultimately the formation of diabetic nephropathy. Circ_0037128 can also up-regulate the expression of KLF9 by sponge binding miR-31-5p. KLF9 is one of the zinc finger proteins involved in gene transcriptional regulation, which is widely involved in the proliferation and differentiation of glomerular cells 38. In addition, KLF9 plays a key role in regulating hepatic glucose metabolism and can activate the process of gluconeogenesis to increase hepatic glucose levels. Therefore, its high expression may be an important risk factor for diabetes and diabetic nephropathy. hsa_Circ_0037128 inhibits podocyte survival by regulating the miR-31-5p/KLF9 axis, thereby promoting apoptosis of podocytes while activating inflammatory and oxidative stress responses. Therefore, knockout of hsa_Circ_0037128 can attenuate hyperglycemia-induced podocyte injury. At the same time inhibit the proliferation of mesangial cells and fibrosis. This suggests that hsa_Circ_0037128 may be a potential target for the treatment of diabetic nephropathy 38. CIRC_0123996 inhibits the proliferation and fibrosis of glomerular extracellular matrix through CIRC_0123996/miR-149-5p/Bach1, and also plays an important regulatory role in diabetic nephropathy. CircEIG4G2 can regulate the formation of transforming growth factor type I collagen and fibronectin induced by hyperglycemia through the mechanism of miR218/ β1, which leads to the formation of fibrosis in renal tubular epithelial cells of DKD 39.

Because the abnormal function of renal tubular epithelial cells (RTECs) and endothelial cells is the key link leading to diabetic nephropathy, its apoptosis can lead to abnormal renal tubular reabsorption and renal fibrosis, thus accelerating the progress of diabetic nephropathy. Therefore, to find out the specific mechanism of RTECs damage and reduce RTECs damage is an important way to reduce the progress of DKD. CircRNA participates in the occurrence and development of diabetic nephropathy by regulating RTECs damage. For example, CircHIPK3 can promote the proliferation of RTECs induced by high glucose and inhibit the progression of diabetic nephropathy through antioxidation, anti-apoptosis and anti-inflammation 40. The main mechanism is to increase the expression of SIRT1 through sponge miR-326/miR-487a-3p/SIRT1, reduce the toxicity of high glucose on renal tubular epithelial cells, inhibit the proliferation of renal tubular epithelial cells, reduce the secretion of inflammatory cytokines and delay the progression of diabetic nephropathy. CIRC_0003928 is the sponge of miR506-3p, and hDAC4 is identified as the target gene of miR-506-3p. HDAC4 is highly expressed in diabetic nephropathy, which is the main factor of podocyte injury and participates in the progression of diabetic nephropathy. Knockout of CIRC_0003928 through miR-506-3p/HDAC4 pathway can improve the proliferation, apoptosis and oxidative stress of RTECs induced by DKD high glucose. It also suggests that CIRC_0003928 may be a potential therapeutic target for DKD 41. Circ_0000064 is the sponge of miR-532-3P, ROCK1 is the target of miR-532-3p, and its expression is suppressed by Circ_0000064 gene knockout. miR-532-3p is significantly downregulated in many kidney diseases including diabetic nephropathy, and its expression may be associated with chronic inflammation, oxidative stress, and the activity of apoptotic pathways. ROCK1 is a protein serine / threonine kinase, which has been shown to be involved in a variety of intracellular biological functions. ROCK1 can promote the interstitial transformation of glomerular endothelial cells and increase proteinuria, thus aggravating the progression of diabetic nephropathy. Circ_0000064 knockout may attenuate hyperglycemia-induced RTECs damage by regulating the miR-532-3p/ROCK1 axis. It also inhibits hyperglycemia-induced oxidative stress, inflammation, apoptosis and extracellular matrix accumulation in mesangial cells, thereby inhibiting mesangial cell proliferation and renal fibrosis. Therefore, CIRC_0000064 may be a key regulatory factor in promoting the progression of diabetic nephropathy and provide a new target for the treatment of DKD 42, 43.

Overexpression of Circ_LARP6 regulates the proliferation of Mesangial cells in diabetic nephropathy by inhibiting the expression of miR-424, and inhibits renal fibrosis by promoting apoptosis 44. CIRC_WBSCR17 participates in the progression of DKD by increasing inflammatory response, cell proliferation, apoptosis and fibrin expression through the miR-185-5p/SOX6 gene axis. CIRC-AKT3 inhibits the proliferation of glomerular Mesangial cells and the production of Mesangial matrix in patients with diabetic nephropathy by regulating miR-296-3phammer E-cadherin signal, thus delaying the development of diabetic nephropathy 45. CIRCDLGAP4 regulates the ERBB3/NF-κB/MMP2 gene axis by activating miR-143. At the same time, it promotes the proliferation and fibrosis of the glomerular extracellular matrix, thereby inducing the progression of diabetic nephropathy. hsa_circ_0068087 is a circular RNA derived from human G protein β4 subunit (GNB4). miR-23c can inhibit hyperglycemia-induced proliferation of mesangial cells, secretion of ECM proteins, and release of pro-inflammatory factors. These results suggest that miR-23c has a potential inhibitory effect on Mesangial cell proliferation, inflammation and oxidative stress induced by hyperglycemia. Early growth response protein-1 (EGR1) is a transcription factor that plays a key role in diabetes and diabetic nephropathy. Interfering hsa_circ_0068087 (circ-GNB4) regulates glomerular Mesangial cell proliferation, ECM accumulation, oxidative stress and inflammation through Circ-GNB4/miR23c/EGR1 pathway, thus alleviating the injury of glomerular Mesangial cells induced by high glucose. This indicates that circ-GNB4 can be used as a potential therapeutic target for intervention in diabetes and the progres sion of DKD 46.

The above CircRNAs results will provide new biomarkers and therapeutic targets for the treatment of diabetic nephropathy. In the past, the diagnosis of diabetic nephropathy generally depends on urinary albumin, serum creatinine, glomerular filtration rate and renal biopsy. CircRNA is helpful for the early diagnosis of diabetic nephropathy because it is located in the cytoplasm or stored in the exocrine body. It is generally not affected by RNA exonuclease, is not easy to degrade, and its expression is very stable. It is helpful for the timely diagnosis of diabetic nephropathy and has greater research prospects. In addition, we also found that up-regulated CircRNAs is more than down-regulated CircRNAs, which indicates that it is important for DKD to study the mechanism of promoting DNA repair.

## Conclusion and future prospects

CircRNA is a new non-coding ribonucleic acid, which can be specifically expressed in tissue/development stage, and has a high degree of stability and incompatibility.The CeRNA mechanism is a typical CircRNA regulation mechanism. CircRNA contains a specific binding site to miRNA, which inhibits its further binding to the target RNA by binding to miRNA. This increases the expression of related genes and plays an important biological role. Recent studies have shown that CircRNA is associated with diabetes, diabetic cardiomyopathy, endothelial dysfunction, atherosclerosis, retinopathy, nerve conduction diseases and so on. Other studies have found that CircRNA plays an important role in inflammation, apoptosis, autophagy, Mesangial cell proliferation, extracellular matrix accumulation, tubulointerstitial fibrosis and sclerosis in diabetic nephropathy. In this paper, the research progress of CircRNA involved in the pathogenesis of diabetic nephropathy through ceRNA process was reviewed, and the CircRNAs expression profile of DKD was reported. We conclude that CircRNA plays an important role as a marker in DKD. Therefore, it can be used as a biomarker for the diagnosis of diabetic nephropathy and a new therapeutic target. However, there are some limitations in the review of diabetic nephropathy. For example, previous studies have been limited to animal experiments, and the number of study cases is relatively small. At present, there are few studies on CircRNA in DKD, so the role of CircRNA in diabetic nephropathy is a subject of concern, which must be solved in future research.

## Author Contributions

C TU and LZ WANG prepared and wrote the manuscript; ZY JIANG and L WEI revised the manuscript.
